# Ladungsunterstützte Selbstmetallierung von Porphyrinen auf Oxidoberflächen

**DOI:** 10.1002/ange.202015187

**Published:** 2021-01-14

**Authors:** Larissa Egger, Michael Hollerer, Christian S. Kern, Hannes Herrmann, Philipp Hurdax, Anja Haags, Xiaosheng Yang, Alexander Gottwald, Mathias Richter, Serguei Soubatch, F. Stefan Tautz, Georg Koller, Peter Puschnig, Michael G. Ramsey, Martin Sterrer

**Affiliations:** ^1^ Institute of Physics NAWI Graz University of Graz Universitätsplatz 5 8010 Graz Österreich; ^2^ Peter Grünberg Institute (PGI-3) Forschungszentrum Jülich 52425 Jülich Deutschland; ^3^ Jülich Aachen Research Alliance (JARA) Fundamentals of Future Information Technology 52425 Jülich Deutschland; ^4^ Experimentalphysik IV A RWTH Aachen University 52074 Aachen Deutschland; ^5^ Physikalisch-Technische Bundesanstalt (PTB) 10587 Berlin Deutschland

**Keywords:** Dünne Filme, Grenzflächen, Ladungstransfer, Metallierung, Porphyrine

## Abstract

Das Interesse an Metallierungs‐ und Selbstmetallierungsreaktionen auf Oxidoberflächen ist in jüngster Zeit ständig gewachsen. Der Mechanismus der Selbstmetallierungsreaktion ist jedoch nicht vollständig geklärt. Hier zeigen wir mithilfe von Rastertunnelmikroskopie, Photoemissions‐Spektroskopie und Dichtefunktionaltheorie‐Rechnungen, dass die Selbstmetallierung von 2H‐Tetraphenylporphyrin auf der Oberfläche von ultradünnen MgO(001)‐Filmen durch Ladungstransfer ermöglicht wird. Es wird gezeigt, dass der Ladungszustand und dadurch der Metallierungszustand der Porphyrin‐Moleküle durch die Austrittsarbeit des MgO(001)/Ag(001)‐Substrats gezielt eingestellt werden können.

## Graphical abstract

Es wird angenommen, dass die Selbstmetallierung von 2*H*‐Tetraphenylporphyrin nur an Defektzentren der MgO‐Oberfläche möglich ist. Hier wird gezeigt, dass die Reaktion durch Ladungstransfer begünstigt wird und nicht notwendigerweise die Präsenz von Defekten erfordert. Durch die Variation der Austrittsarbeit von ultradünnen MgO‐Filmen als Substraten ist es möglich, den Ladungs‐ und Metallierungszustand der darauf adsorbierten Moleküle zu steuern.

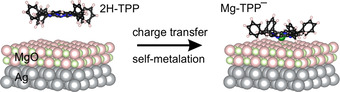

Porphyrine und Metalloporphyrine spielen aufgrund ihrer vielseitig variierbaren Eigenschaften eine zentrale Rolle in vielen Bereichen von organisch‐anorganischen Hybrid‐Materialien. Speziell die oberflächenvermittelten Prozesse von und mit Porphyrinen haben zur ungebrochenen Faszination dieser Materialien beigetragen.^[1]^ Zum grundlegenden Verständnis von ihren chemischen und physikalischen Eigenschaften haben vor allem oberflächenphysikalische Untersuchungen beigetragen. Dazu zählen unter anderem Studien zur Selbstorganisation, zur On‐Surface‐Synthese von kovalent verbundenen Metall‐organischen 2D‐Netzwerken und Herstellung von 3D‐Gerüststrukturen^[2]^ sowie zur gezielten Metallierung^[3]^ und Ligation, um die magnetischen, sensorischen und katalytischen Eigenschaften zu steuern.^[4]^

Die Porphyrin‐Metallierung an Oberflächen wird typischerweise durch Prä‐ oder Postdeponierung von Metall‐Adatomen oder durch Selbstmetallierung, die z. B. auf Substraten wie Cu, Ni and Fe stattfindet, erreicht.^[3a]^ Diese Redoxreaktionen, welche konformative Intermediate, Wasserstoff‐Transfer‐Prozesse und schlussendlich Wasserstoff‐Abgabe beinhalten, sind gut verstanden.^[5]^ Selbstmetallierungsreaktionen wurden in jüngster Zeit auch auf einkristallinen Oxidsubstraten, z. B. auf TiO_2_(110)^[6]^ und dünnen Co_*x*_O_*y*_ ^[7]^‐ und MgO(001)^[8]^‐Filmen, beobachtet. Im Gegensatz zur Metallierung mit Metall‐Adatomen kann die Selbstmetallierung auf Oxiden als Ionenaustausch‐Prozess beschrieben werden, in dem die Protonen der freien Porphyrin‐Base durch ein Metall‐Kation ersetzt werden. Die genauen mechanistischen Aspekte dieser Reaktionen sind jedoch kaum ergründet. Hier liefern wir einen überzeugenden Beweis dafür, dass die Selbstmetallierung von 2*H*‐Tetraphenylporphyrin (2H‐TPP) auf der Oberfläche von ultradünnen MgO(001)‐Filmen durch Ladungstransfer ermöglicht wird. Zudem zeigen wir, dass über die Kontrolle der Substrat‐Eigenschaften die gezielte Stabilisierung von ungeladenen/nicht‐metallierten und geladenen/metallierten Molekülen erreicht werden kann.

Die bei der Selbstmetallierungsreaktion von Porphyrinen auf Oxidoberflächen freigesetzten Protonen führen zur Oberflächen‐Hydroxylierung.[^8^, ^9^] Im Fall der Selbstmetallierung von 2H‐TPP auf MgO hat die Hydroxylierung einen bedeutenden Anteil, da erst durch sie die Reaktion thermodynamisch ermöglicht wird.^[8]^ Dies hängt jedoch stark von der Morphologie ab, und Untersuchungen von MgO‐Nanowürfeln zeigten, dass die Reaktion auf niedrig‐koordinierte Zentren wie Kanten und Ecken, wo die Bildungsenergie von Mg^2+^‐Fehlstellen niedriger ist und die Hydroxygruppen stabiler sind als auf MgO‐Terrassen, limitiert ist.[^8^, ^9b^] Im Gegensatz zu diesen Studien stehen die Ergebnisse mit ultradünnen MgO‐Filmen, wo die Metallierung einer gesamten 2H‐TPP‐Monolage beobachtet wurde.^[9a]^ Während dünne MgO‐Filme natürlich auch Defekte enthalten,^[10]^ kann deren limitierte Konzentration jedoch nicht für dieses Ergebnis verantwortlich sein, weshalb die besondere Selbstmetallierungsaktivität auf ultradünnen MgO‐Filmen weiterhin mysteriös bleibt. Es scheinen zusätzliche Parameter für diesen Effekt verantwortlich zu sein, deren Entschlüsselung detailliertes Wissen und Verständnis der morphologischen und elektronischen Eigenschaften des kombinierten Molekül‐Substrat‐Systems verlangen, das wir hier mithilfe einer Kombination von Rastertunnelmikroskopie (STM)‐ und Photoemissions‐Spektroskopie‐Experimenten und Rechnungen mit Dichtefunktionaltheorie (DFT) erlangen (Hintergrundinformationen SI.1, SI.2).

STM‐Bilder der Oberfläche des dünnen MgO(001)‐Films vor und nach der Adsorption von 2H‐TPP sind in Abbildung 1 a,b dargestellt. Wie weiter unten gezeigt wird, bestätigen unsere Röntgen‐Photoelektronen‐Spektroskopie (XPS)‐Ergebnisse, dass es zur spontanen Metallierung der 2H‐TPP‐Monolage kommt, weshalb angenommen werden kann, dass die meisten der abgebildeten Porphyrinmoleküle bereits im metallierten Zustand, Mg‐TPP, sind. Die Moleküle sind in einer hochgeordneten quadratischen Phase mit zwei Rotationsdomänen angeordnet. Dies ist auch anhand des entsprechenden Beugungsmusters ersichtlich, welches in den Hintergrundinformationen abgebildet ist und als $\left(\begin{array}{cc} 4 & -2\\ 2 & 4 \end{array}\right)$ ‐ Überstruktur mit einer quadratischen Elementarzelle und einem Gittervektor mit 13.3 Å Länge interpretiert werden kann. Detailreichere STM‐Aufnahmen von isolierten Molekülen und von der Monolagenphase sind in Abbildung 2 a,b gezeigt. Die isolierten Moleküle erscheinen als vier im Quadrat angeordnete Erhöhungen mit einer Mulde in der Mitte (Abbildung 2 a). Die vier Erhöhungen werden den Phenylgruppen zugeordnet, und die gegenüberliegende Phenylgruppen verbindenden Achsen zeigen in die [110]‐Richtungen. Die Identifizierung der einzelnen Moleküle ist weniger eindeutig im Fall der geschlossenen Monolage, wird jedoch durch das Auftreten einer Molekül‐Fehlstelle erleichtert, wie in Abbildung 2 b dargestellt. Dies zeigt, dass in diesem Fall die kreisförmig angeordneten Erhebungen nicht zu den vier Phenylgruppen eines Moleküls, sondern zu den Phenylgruppen von vier benachbarten Molekülen gehören. Die im STM‐Bild gefundene Anordnung der Moleküle ist in perfekter Übereinstimmung mit dem Beugungsbild und führt zum schematischen Oberflächenmodell, das in Abbildung 2 c dargestellt ist. Die gezeigte Anordnung führt zur Maximierung der Stabilität aufgrund der T‐artigen Wechselwirkung zwischen den Phenylringen.^[11]^ Die spezielle Erscheinung der Moleküle in STM deutet auf eine durch die starke Wechselwirkung des Makrozyklus mit dem Substrat hervorgerufene Verkippung und Verdrehung der Phenylringe hin (Abbildung 2 d).^[12]^ Die nach oben verkippten Phenylringe dominieren den Bildkontrast und erlauben es daher nicht, die hauptsächlich am Makrozyklus lokalisierte Orbitalstruktur abzubilden. Diese Information wäre allerdings nötig, um Informationen zur elektronischen Struktur und zum Ladungszustand der Moleküle ableiten zu können. Deshalb wenden wir uns nun der detaillierteren Untersuchung der besetzten elektronischen Zustände mittels Winkel‐aufgelöster Ultraviolett‐Photoemissions‐Spektroskopie (ARUPS) und XPS zu.

**Figure 1:**
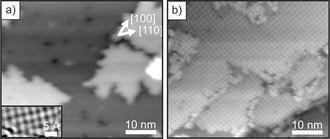
STM‐Bilder von a) 2 ML MgO(001)/Ag(001) (*U*_bias_=3.0 V, *i*_t_=29 pA), b) 1 ML 2H‐TPP auf 2 ML MgO(001)/Ag(001) bei Raumtemperatur adsorbiert und anschließend auf 473 K geheizt (*U*_bias_=2.0 V, *i*_t_=28 pA). Einschub in (a): atomar aufgelöstes Bild der MgO(001)‐Oberfläche.

**Figure 2:**
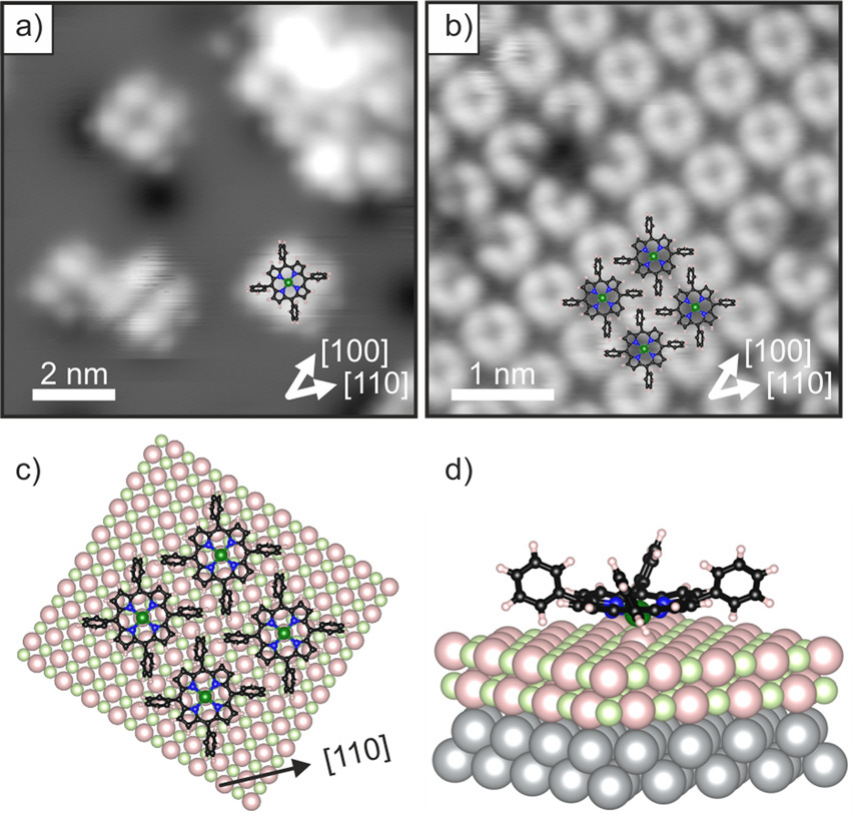
STM‐Bilder a) eines 2H‐TPP‐Films niedriger Bedeckung, der bei 80 K auf 2 ML MgO(001)/Ag(001) aufgebracht wurde (*U*_bias_=1.2 V, i_t_=25 pA), und b) einer geordneten Monolagen‐Schicht (*U*_bias_=3.2 V, *i*_t_=55 pA). c) Aufgrund von STM und LEED abgeleitetes Strukturmodell der Monolagenphase (Draufsicht). d) 3D‐Ansicht des mit DFT erhaltenen Adsorptionsmodells von negativ geladenem Mg‐TPP auf 2 ML MgO(001)/Ag(001).

Die ARUPS‐ und N 1s‐XP‐Spektren von der sauberen MgO(001)/Ag(001)‐Oberfläche sowie von zunehmend größeren Dosierungen von 2H‐TPP auf dieser Oberfläche sind in Abbildung 3 gezeigt. In den XP‐Spektren erkennen wir nach Adsorption einer Monolage 2H‐TPP einen einzelnen N 1s‐Peak bei einer Bindungsenergie (BE) von 399 eV, was darauf hindeutet, dass alle 4 N‐Atome die gleiche chemische Umgebung besitzen, wie es in metalliertem TPP der Fall ist.^[13]^ Dieser Peak bleibt bei weiterer Dosierung von 2H‐TPP erhalten, während ein Paar von zusätzlichen N 1s‐Peaks bei 400.5 eV und 398 eV BE, welche den Amin‐ und Imin‐Stickstoffpaaren in nicht‐metalliertem 2H‐TPP zugeordnet werden können,^[13]^ bei weiterer Adsorption von 2H‐TPP hinzukommt. Dieses Ergebnis bestätigt frühere Untersuchungen und weist darauf hin, dass die Moleküle der Monolage zu Mg‐TPP metalliert werden, während die Moleküle der zweiten und aller darauffolgenden Lagen unmetalliert bleiben.^[9a]^ An dieser Stelle soll noch einmal erwähnt sein, dass die hohe Konzentration von metallierten TPP‐Molekülen auf den dünnen MgO‐Filmen zusammen mit der flachen, terrassenartigen Morphologie der Filme nicht mit einer Defekt‐getriebenen Selbstmetallierungsreaktion, wie in früheren Arbeiten propagiert, vereinbar ist (Hintergrundinformationen, SI.3).[^8^, ^9b^] Es muss deshalb eine andere Triebkraft vorhanden sein, die zu dieser Reaktion auf den dünnen MgO‐Filmen führt.

**Figure 3:**
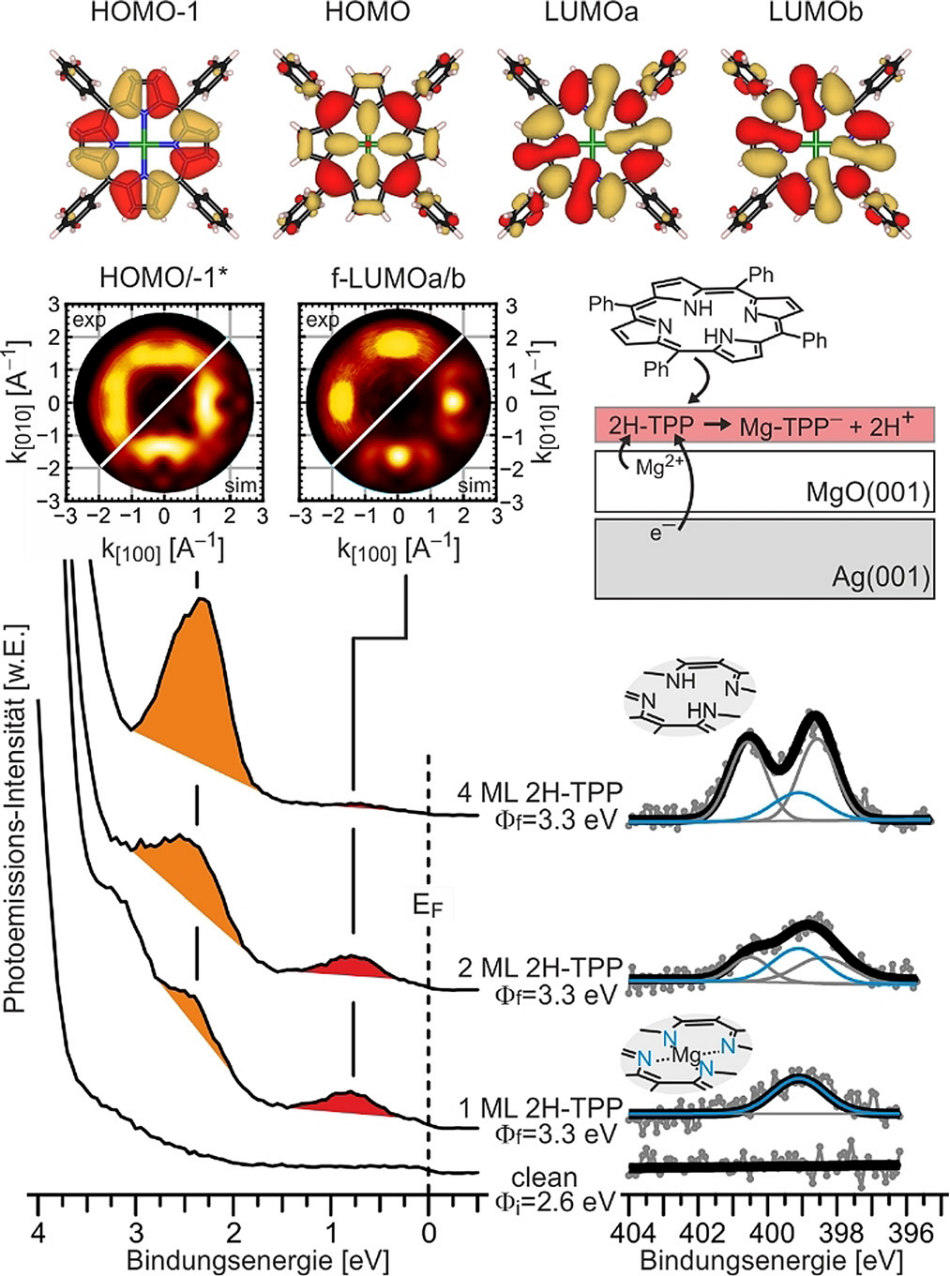
Oben: Realraum‐Darstellung des HOMO−1, HOMO und der entarteten LUMOa und LUMOb von Gasphasen‐Mg‐TPP. ARUPS‐ (unten links) und XPS‐Spektren (unten rechts) von 2 ML MgO(001)/Ag(001) und zunehmend größer werdenden Dosierungen von 2H‐TPP (1, 2 und 4 ML) auf 2 ML MgO(001)/Ag(001). *Φ*_i_ ist die Austrittsarbeit von MgO(001)/Ag(001), und *Φ*_f_ ist die Austrittsarbeit nach Adsorption von 2H‐TPP. Mitte, links: Vergleich von experimentell gemessenen und simulierten Impulsraumverteilungen von 1 ML 2H‐TPP auf MgO(001)/Ag(001). Die experimentellen Daten wurden bei Bindungsenergiewerten entsprechend den Maxima der PE‐Peaks bei 0.75 und 2.3 eV erhalten und zeigen die Überlagerung von LUMOa/b (0.75 eV) und von HOMO und HOMO−1 (bei 2.3 eV). Mitte, rechts: Schematisches Modell des Ladungstransfers und der Metallierung bei der Adsorption von 2H‐TPP auf MgO(001)/Ag(001).

Es ist erwiesen, dass die Deponierung von MgO‐Filmen auf Ag(001) zu einer deutlichen Senkung der Austrittsarbeit von Ag(001) führt, wodurch der Ladungstransfer in Adsorbate mit entsprechend hoher Elektronenaffinität (EA) begünstigt wird.^[14]^ 2H‐TPP besitzt eine ähnlich hohe EA (1.6–1.8 eV)^[15]^ wie Pentacen, welches, wie früher gezeigt, auf einer MgO(001)/Ag(001)‐Oberfläche negativ geladen wird.^[16]^ Dass es auch im vorliegenden Fall zu einem Ladungstransfer in die 2H‐TPP‐Moleküle kommt, wird anhand der in Abbildung 3 gezeigten ARUPS‐Ergebnisse deutlich. Die Adsorption von 2H‐TPP auf 2 ML MgO(001)/Ag(001) mit einer anfänglichen Austrittsarbeit von *Φ*_i_=2.6 eV führt zu neuen Zuständen innerhalb der Bandlücke von MgO, die den Grenzorbitalen von 2H‐TPP zugeordnet werden können. Zwei Emissionen bei 0.75 eV und 2.3 eV BE sind unmittelbar nach der Adsorption einer 2H‐TPP‐Monolage zu erkennen. Gleichzeitig kommt es zu einer Erhöhung der Austrittsarbeit auf 3.3 eV, was ein erster Hinweis für einen Ladungstransfer in die adsorbierten Moleküle ist.^[17]^ Wenn die Molekül‐Bedeckung auf 2 ML und 4 ML erhöht wird, kommt es zu einer Abschwächung des 0.75‐eV‐Signals, während die Intensität der Emission bei 2.3 eV BE weiter ansteigt. Dadurch wird gezeigt, dass das erste Signal ausschließlich von Molekülen aus der Monolage stammt. Da die zusätzlichen Moleküllagen zu keiner weiteren Austrittsarbeitsänderung führen, kann auch geschlossen werden, dass der Ladungstransfer auf die interfaciale Monolage beschränkt ist.

Die Identifizierung der Molekülorbitale, aus welchen die photoemittierten Elektronen stammen, wird durch eine als Photoemissionstomographie bekannte Methode ermöglicht. Hierbei wird die Winkelverteilung der Intensität der photoemittierten Elektronen aufgenommen und in den Impulsraum konvertiert. Die so erhaltene zweidimensionale Impulsraumverteilung kann annähernd als reziprokes Bild der Elektronenverteilung im Realraum interpretiert werden.^[18]^ Die experimentell erhaltenen Impulsraumverteilungen der beiden Molekülemissionen bei den jeweiligen Intensitätsmaxima sind zusammen mit den simulierten Verteilungen der entarteten TPP‐LUMOs (für die Emission bei 0.75 eV) und für die Überlagerung von HOMO und HOMO−1 (für die Emission bei 2.3 eV) in Abbildung 3 dargestellt. Die entsprechenden Realraum‐Darstellungen der Molekülorbitale sind in Abbildung 3 (oben) gezeigt. HOMO und HOMO−1 liegen energetisch zu nahe beisammen, als dass sie im gezeigten Experiment getrennt aufgelöst werden könnten. Anhand der guten Übereinstimmung kann der Peak bei 0.75 eV der Emission eines besetzten Zustandes, der die Elektronendichteverteilung des molekularen LUMO besitzt, zugeordnet werden. Dadurch ist es nun auch eindeutig belegt, dass es zu einem Ladungstransfer während der Adsorption von 2H‐TPP auf MgO(001)/Ag(001) kommt, und der entsprechende Zustand wird fortan als früheres LUMO (f‐LUMO) bezeichnet. Da der Ladungstransfer durch die dünne MgO‐Schicht durch Elektronentunneln erklärt werden kann, ist zusätzlich festzuhalten, dass der Zustand ein “integer charge transfer”‐Zustand ist.[^16^, ^17^]

Anhand der gemeinsamen XPS/ARUPS‐Ergebnisse schließen wir, dass während der Adsorption von 2H‐TPP auf ultradünnen MgO‐Filmen zwei Prozesse simultan stattfinden: 1) ein ganzzahliger Ladungstransfer in die Moleküle durch Elektronentunneln und 2) eine Selbstmetallierungsreaktion zu Mg‐TPP. Ob die beiden Prozesse unabhängig voneinander sind oder ob ein Prozess die Voraussetzung für den anderen ist, kann anhand der in Abbildung 3 dargestellten Ergebnisse noch nicht eindeutig geklärt werden. Um das Zusammenspiel von Ladungstransfer und Metallierung zu zeigen, wenden wir deshalb eine erst kürzlich entwickelte Strategie zur Unterbindung des Ladungstransfers an.^[17]^ Diese basiert auf der chemischen Modifizierung der MgO/Ag‐Grenzfläche durch Magnesium bzw. Sauerstoff und erlaubt es, die Austrittsarbeit in einem weiten Bereich, zwischen 2.3 eV und 4.4 eV, zu variieren (Hintergrundinformationen, SI.3). Wenn es zum Ladungstransfer kommt, entspricht die nach Sättigung der Monolage erhaltene Austrittsarbeit der kritischen Austrittsarbeit für den Ladungstransfer (Hintergrundinformationen, SI.4). Im vorliegenden Fall (Abbildung 3) liegt die kritische Austrittsarbeit bei *Φ*_crit_=3.3 eV. Es ist deshalb anzunehmen, dass der Ladungstransfer in adsorbierte 2H‐TPP‐Moleküle unterbunden wird, wenn sie auf einem MgO(001)/Ag(001)‐Substrat mit *Φ*>3.3 eV adsorbiert werden.

In Abbildung 4 a,b vergleichen wir die Photoemissionsspektren von 2H‐TPP‐Monolagen, die auf MgO(001)/Ag(001)‐Filmen unterschiedlicher Austrittsarbeit, einmal unterhalb *Φ*_crit_ (2.6 eV) und einmal oberhalb *Φ*_crit_ (3.9 eV), adsorbiert wurden. Die Spektren von 2H‐TPP auf dem Substrat mit niedriger Austrittsarbeit spiegeln die Ergebnisse aus Abbildung 3 wider, wo anhand der Beobachtung des f‐LUMO in ARUPS und des einzelnen N 1s‐XP‐Peaks der Ladungstransfer und die Selbstmetallierung nachgewiesen wurden. Wenn jedoch 2H‐TPP auf dem Substrat mit hoher Austrittsarbeit adsorbiert wird, kommt es nicht zur Bildung eines neuen Zustands in der Bandlücke, wodurch bestätigt wird, dass hier kein Ladungstransfer stattfindet. Zusätzlich ist nun im XP‐Spektrum die typische Signatur von nicht‐metalliertem 2H‐TPP zu erkennen.

**Figure 4:**
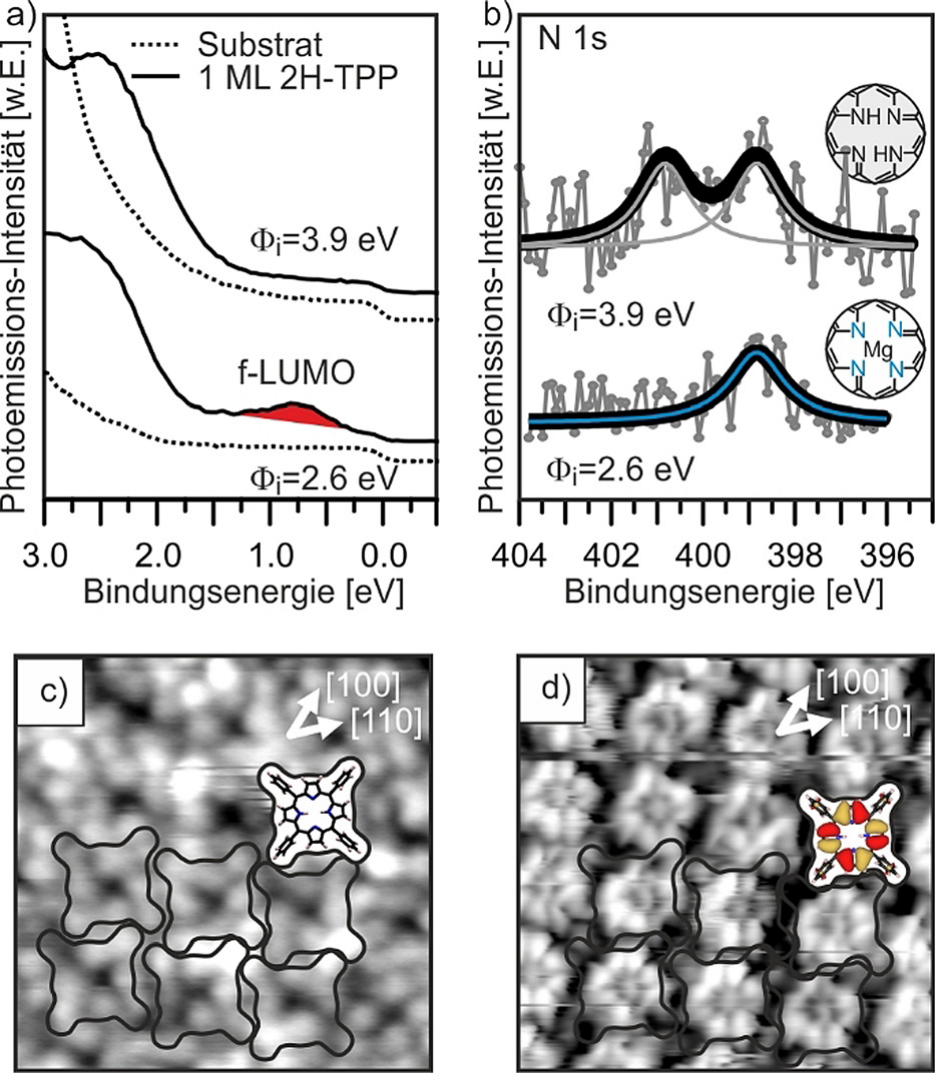
ARUPS (a) und N 1s‐XPS (b) von einer auf 2 ML MgO(001)/Ag(001) mit unterschiedlichen anfänglichen Austrittsarbeiten *Φ*_i_ adsorbierten 2H‐TPP Monolage. c,d) STM bei *U*_bias_=+2.9 V (c) und *U*_bias_=−2.6 V (d) von 2H‐TPP, welches auf einem MgO(001)/Ag(001)‐Film mit hoher anfänglicher Austrittsarbeit adsorbiert wurde.

Der nicht stattgefundene Ladungstransfer wirkt sich auch auf die Erscheinung der Moleküle in der STM aus. Anstelle der durch die Phenylringe dominierten Erscheinung wie in Abbildung 2 können bei gleichen Tunnelbedingungen sowohl die Phenylringe als auch der Makrozyklus abgebildet werden (Abbildung 4 c). Darüber hinaus konnte bei negativer Tunnelspannung submolekularer Kontrast, entsprechend der Elektronendichteverteilung des HOMO−1, erreicht werden. Zusätzlich ist noch zu erkennen, dass die 2H‐TPP‐Moleküle in den STM‐Bildern in Abbildung 4 im Vergleich zu jenen in Abbildung 1 und 2 um 45° gedreht sind, d. h., die Phenylachse ist nun entlang der [100]‐Substratrichtungen ausgerichtet. Der unterschiedliche STM‐Kontrast lässt darauf schließen, dass die 2H‐TPP‐Moleküle flacher sind und dass es im Gegensatz zu den geladenen Molekülen zu keiner Verkippung und Verdrehung der Phenylgruppen kommt. Das impliziert eine schwächere Wechselwirkung der ungeladenen 2H‐TPP‐Moleküle mit dem Substrat im Vergleich zu den geladenen und metallierten Molekülen, was sich auch auf die Stabilität der Monolagen‐Phase auswirkt: Während die geladene und metallierte Monolage bis zumindest 473 K stabil ist (Abbildung 1 b), kommt es bei der ungeladenen und unmetallierten Monolage bei gleicher thermischer Behandlung zu starken strukturellen Veränderungen und Desintegration der Monolage.

Mithilfe der oben vorgestellten Ergebnisse konnten wir zeigen, dass die Selbstmetallierungsreaktion von 2H‐TPP auf ultradünnen MgO‐Filmen durch Ladungstransfer von der MgO/Ag‐Grenzfläche in das LUMO des 2H‐TPP begünstigt wird. Beachtenswert ist dabei, dass dieser Prozess nicht auf Defekte wie niedrig‐koordinierte Zentren an Ecken und Kanten limitiert ist, sondern auch auf defektfreien Terrassen stattfindet. Wie in früheren Arbeiten gezeigt wurde, ist die Selbstmetallierungsreaktion auf den Terrassen von MgO‐Volumenkristallen energetisch nicht möglich, da der Energiegewinn aufgrund der Hydroxylierung nicht ausreicht, um den hohen energetischen Aufwand zur Bildung der Mg^2+^‐Fehlstelle zu kompensieren.^[8]^ Offensichtlich ist der Ladungstransfer in die 2H‐TPP‐Moleküle auf den dünnen MgO‐Filmen dafür verantwortlich, dass die Metallierungsreaktion auf den Terrassenplätzen thermodynamisch begünstigt wird. Dazu tragen als Begleiterscheinung zur Ladung die stärkere Wechselwirkungsenergie und der reduzierte Molekül‐Substrat‐Abstand bei. Zusätzlich führt die Anwesenheit von geladenen Molekülen zu signifikanten Atom‐Verschiebungen im MgO‐Film, was die Selbstmetallierungsreaktion durch Erniedrigung der Fehlstellen‐Bildungsenergie zusätzlich befördern kann.

Zum Abschluss weisen wir darauf hin, dass die hier vorgestellten Ergebnisse auch eine rationale Erklärung für die anscheinend widersprüchlichen Ergebnisse von früheren Adsorptionsstudien von 2H‐TPP auf dünnen MgO‐Filmen liefern. Dabei wurde gefunden, dass auf 10 ML dünnen MgO‐Filmen 50 % der 2H‐TPP‐Moleküle metalliert sind,^[8]^ während auf 2 ML dünnen Filmen vollständige Metallierung stattfindet.^[9a]^ Dieses Ergebnis lässt sich anhand der Elektrostatik des Systems erklären. Der Ladungstransfer ist eine Konsequenz des Potenzialausgleichs im kombinierten Adsorbat‐Substrat‐System, welcher im vorliegenden Fall mit einem einfachen Plattenkondensator‐Modell beschrieben werden kann.^[17]^ In diesem Modell muss es aufgrund der Konstanz des Potenzials bei einer Vergrößerung der Schichtdicke zu einer Abnahme der transferierten Ladung kommen. Deshalb sind auf dickeren MgO‐Schichten weniger geladene und deshalb auch weniger metallierte Moleküle vorhanden (Hintergrundinformationen, SI. 4). Es sei noch angemerkt, dass die im Rahmen dieser Arbeit durchgeführten DFT‐Rechnungen den Ladungstransfer in die 2H‐TPP‐Moleküle und die damit verbundenen Austrittsarbeitsänderungen zufriedenstellend beschreiben. Jedoch konnte in Bezug auf die Selbstmetallierungsreaktion selbst mit der Berücksichtigung von Van‐der‐Waals‐Korrekturen keine gute Übereinstimmung mit dem Experiment erreicht werden (Hintergrundinformationen, SI.5). Bei der Selbstmetallierungsreaktion von 2H‐TPP auf der MgO(001)/Ag(001)‐Oberfläche handelt es sich um eine komplexe Reaktion, die ionische Bindung, schwache Physisorption und Ladungstransfer vom Metall ins Moleküle beinhaltet. Diese Effekte können mit Ab‐initio‐Methoden individuell gut beschrieben werden, stellen aber zusammen eine große Herausforderung und eine noch zu bearbeitende Aufgabe für zukünftige Rechnungen dar.

Hier haben wir gezeigt, dass die hohe 2H‐TPP‐Selbstmetallierungsaktivität auf ultradünnen MgO(001)/Ag(001)‐Filmen auf den Ladungstransfer in die 2H‐TPP‐Moleküle zurückzuführen ist. Dieses Ergebnis liefert wichtige Hinweise für den Mechanismus der Selbstmetallierung von Porphyrinen auf Oxidoberflächen. Die aufgrund des Ladungstransfers verursachten konformativen Änderungen im Molekül sowie die Reduktion des Molekül‐Substrat‐Abstands und die verstärkten Atomverschiebungen auf der MgO‐Oberfläche tragen zusammen zu einer positiven Beeinflussung des Reaktionswegs bei. Die Ergebnisse dieser Arbeit zeigen außerdem eine Methode zur Kontrolle der elektronischen und chemischen Zustände von Porphyrinen (geladen und metalliert vs. ungeladen und unmetalliert) über die Variation der Austrittsarbeit des Substrats bzw. die Dicke des Dielektrikums auf, wodurch der Möglichkeit zur selektiven Oberflächenfunktionalisierung der Weg geebnet wird.

Rohdaten sind im öffentlichen Repository Jülich DATA verfügbar.^[19]^

## Conflict of interest

Die Autoren erklären, dass keine Interessenkonflikte vorliegen.

## Supporting information

As a service to our authors and readers, this journal provides supporting information supplied by the authors. Such materials are peer reviewed and may be re‐organized for online delivery, but are not copy‐edited or typeset. Technical support issues arising from supporting information (other than missing files) should be addressed to the authors.

Supplementary
